# Modified BuShenYiQi formula alleviates experimental allergic asthma in mice by negative regulation of type 2 innate lymphoid cells and CD4^+^ type 9 helper T cells and the VIP–VPAC2 signalling pathway

**DOI:** 10.1080/13880209.2021.1970198

**Published:** 2021-09-07

**Authors:** Muhua Huang, Jinfeng Wu, Jingcheng Dong

**Affiliations:** a Department of Integrative Medicine, Huashan Hospital, Fudan University, Shanghai, China; b Institute of Integrative Medicine, Fudan University, Shanghai, China

**Keywords:** Chinese medicine, type 2 immune response, neuro-immune communication, airway inflammation

## Abstract

**Context:**

Modified BuShenYiQi formula (M-BYF) is derived from BuShenYiQi formula, used for the treatment of allergic asthma. The exact effect and mechanism of M-BYF on the improvement of asthma remain unclear.

**Objective:**

We investigated the mechanism underlying the therapeutic effect of M-BYF on allergic asthma.

**Materials and methods:**

The asthma model was established in female BALB/c mice that were sensitized and challenged with ovalbumin (OVA). Mice in the treated groups were orally treated once a day with M-BYF (7, 14 and 28 g/kg/d) or dexamethasone before OVA challenge. Control and Model group received saline. Pathophysiological abnormalities and percentages of lung type 2 innate lymphoid cells (ILC2s) and Th9 cells were measured. Expression levels of type 2 cytokines and transcription factors required for these cells function and differentiation were analysed. Expression of vasoactive intestinal polypeptide (VIP)–VPAC2 signalling pathway-related proteins, and percentages of VIP expressing (VIP^+^) cells and VPAC2, CD90 co-expressing (VPAC2^+^CD90^+^) cells were detected.

**Results:**

M-BYF alleviated airway hyperresponsiveness, inflammation, mucus hypersecretion and collagen deposition in asthmatic mice. M-BYF down-regulated percentages of ILC2s and Th9 cells with lower expression of GATA3, PU.1 and IRF4, reduced IL-5, IL-13, IL-9 and VIP production. The decrease in the expression of VIP–VPAC2 signalling pathway and percentages of VIP^+^ cells, VPAC2^+^CD90^+^ cells were observed after M-BYF treatment. The LD_50_ value of M-BYF was higher than 90 g/kg.

**Discussion and conclusions:**

M-BYF alleviated experimental asthma by negatively regulating ILC2s and Th9 cells and the VIP–VPAC2 signalling pathway. These findings provide the theoretical basis for future research of M-BYF in asthma patient population.

## Introduction

Allergic asthma is a chronic inflammatory disease which affects approximately 300 million people worldwide (Nanda and Wasan 2020). The pathophysiological abnormalities of asthma are airway inflammation, airway hyperresponsiveness (AHR), mucus hypersecretion and airway remodelling, which might form a deleterious positive feedback loop that leads to exaggerated airway narrowing and irreversible loss of lung function (Holgate et al. 2015). Despite the fact that prevalence of asthma continues to rise, its pathophysiological mechanisms are still not fully characterized. New drug discovery has progressed more slowly than in other specialties (Noval Rivas and Chatila 2016; Pavord et al. 2018).

Aberrant type 2 immune responses underlie the pathologies in asthma (Woodruff et al. 2009; Fahy 2015). It is traditionally believed that CD4^+^ type 2 helper T (Th2) cells are the principal drivers of type 2 immune responses. However, recent studies have suggested that type 2 innate lymphoid cells (ILC2s) and CD4^+^ type 9 helper T (Th9) cells play crucial roles in the initiation and exacerbation of type 2 immune responses in asthma (Martinez-Gonzalez et al. 2016; Yu et al. 2018). They are enriched in the blood and tissues of allergic individuals (Jia et al. 2017; Yu et al. 2018; Kerscher et al. 2019). Airway exposure to allergen induces the differentiation and activation of lung ILC2s and Th9 cells to secrete type 2 cytokines including interleukin-13 (IL-13), IL-5 and IL-9, resulting in type 2 airway inflammation, AHR, mucus hypersecretion and structural remodelling (Chen et al. 2013; Beale et al. 2014; Christianson et al. 2015; Li et al. 2016). Moreover, previous studies have implicated the concerted action of ILC2s and Th9 cells as an important cause of pathophysiological abnormalities of asthma (Ying et al. 2016; Moretti et al. 2017).

Neuro-immune communication also plays a critical role in the initiation of potent type 2 immune responses (Voisin et al. 2017). The neuropeptide vasoactive intestinal polypeptide (VIP), which is generated by subsets of cholinergic and sensory nerves, and by some leukocytes, especially T cells after immune activation, induces and promotes type 2 immune responses *in vivo* and *in vitro* (Voice et al. 2003; Huang et al. 2006; Liu et al. 2007). A recent study demonstrated that in a mouse model of OVA-induced allergic inflammation and AHR, activated immune cells produced cytokine IL-5 to directly activate pulmonary sensory neurons. Activated sensory neurons released VIP, which then acted on ILC2s and CD4^+^ T cells via type 2 VIP receptor (VPAC2) to induce cytokines production, creating an inflammatory signalling loop that promoted type 2 airway inflammation (Talbot et al. 2015). VIP–VPAC2 axis on T cells enhances Th2 cells differentiation, and promotes Th2 cell-derived cytokines production inhibiting the production of Th1 cell-derived cytokines (Voice et al. 2001; Delgado and Ganea 2013; Villanueva-Romero et al. 2019). The molecular mechanism of how VIP–VPAC2 signalling modulates the differentiation and function of T cells and ILC2s is still being elucidated. It has been found that VIP interaction with VPAC2 receptor positively regulates intracellular synthesis of cyclic adenosine monophosphate (cAMP) which leads to activation of cAMP-dependent protein kinase A (PKA) (Grinninger et al. 2004; Villanueva-Romero et al. 2019). Activation of cAMP-PKA pathway could induce IL-9 production and GATA binding protein 3 (GATA3), purine-rich box 1 (PU.1) expression in Th9 cells (Mikami et al. 2013). Moreover, enhanced PKA activity could induce Th2 cell-derived cytokine IL-5 gene expression in a GATA3-dependent manner (Klein-Hessling et al. 2003; Hashiguchi et al. 2014). Enforced expression of GATA3 in T cells and ILC2s increases susceptibility to allergic airway inflammation in mice (KleinJan et al. 2014). Hence, modulation of ILC2s and Th9 cells differentiation and function, as well as reduction of VIP–VPAC2 signalling pathway may be potential therapeutic strategies for asthma.

Our previous studies revealed that BuShenYiQi formula (BSYQF) consisting of *Epimedium brevicornu* Maxim., Trudy Imp.S. (Berberidaceae), *Astragalus membranaceus* f. *pallidipurpureus* P.K.Hsiao (Leguminosae) and *Rehmannia glutinosa* f. *lutea* Y.C.Chu & J.F.Li (Scrophulariaceae) could effectively reduce pathophysiological abnormalities of asthma by regulating imbalanced status of helper T cells responses and neuroendocrine function (Wang, Wu et al. 2014; Kong et al. 2015). Moreover, *Scutellaria baicalensis* f. *albiflora* H.W.Jen & Y.J.Chang (Lamiaceae) and *Paeonia lactiflora* var. *villosa* M.S.Yan & K.Sun (Paeoniaceae) have also been reported to effectively inhibit allergic inflammatory responses and ameliorate the progression of asthma (Lee et al. 2008; Bui et al. 2017). It remains unknown whether a combination of these herbs (M-BYF) has a protective effect against asthma. The related mechanisms remain to be determined. In this study, a formulation M-BYF is investigated for its protective efficacy in pathophysiological abnormalities in an OVA-induced mouse asthma model. We explored whether modulation of ILC2s, Th9 cells and VIP–VPAC2 signalling pathway is involved in the treatment effects of M-BYF on asthma.

## Materials and methods

### Reagents

Ovalbumin (OVA) (grade II and V), aluminium hydroxide (Alum), acetyl-β-methacholine (MCh), collagenase IV and DNase I were supplied from Sigma-Aldrich (St. Louis, MO). Dexamethasone was obtained by Chenxin Pharmaceutical Co., Ltd. (Jining, China). Icariin (HPLC ≥98%), calycosin-7-glucoside (HPLC ≥98%), catalpol (HPLC ≥98%), baicalin (HPLC ≥98%), paeoniflorin (HPLC ≥98%) were purchased from Winherb Medical Technology Co., Ltd. (Shanghai, China). Enzyme-linked immunosorbent assay (ELISA) kits for IL-13, IL-5 were purchased from Biolegend (San Diego, CA). The ELISA kit for VIP was obtained from Phoenix Pharmaceuticals (Belmont, CA). Antibodies including eF506-conjugated anti-CD45, PE-conjugated anti-CD90.2, PE-Cy7-conjugated anti-CD3, PE-Cy7-conjugated anti-CD4, PE-Cy7-conjugated anti-CD19, PE-Cy7-conjugated anti-CD11b, eF450-conjugated anti-CD127, APC-conjugated anti-ST2, APC-conjugated anti-IL-9 and APC-Cy7-conjugated Live/Dead were purchased from eBioscience (San Diego, CA). Leukocyte activation cocktail and Foxp3 Permeabilization (Perm)/Fixation buffer were obtained from BD Biosciences (San Diego, CA). The antibody against VPAC2 was obtained from Affinity Biosciences (Cincinnati, OH). The antibodies against β-actin and phospho-(Ser/Thr) PKA substrate were purchased from Cell Signaling Technology (Danvers, MA). The antibodies against VIP, GATA3, cAMP and cluster of differentiation (CD)90 were obtained from Proteintech Group (Wuhan, China). The RIPA lysis buffer, proteinase inhibitor, BCA protein assay kit and RNAeasy mini kit were acquired from Beyotime Institute of Biotechnology (Shanghai, China). The enhanced chemiluminescence (ECL) detection kit was obtained from Millipore Corp. (Temecula, CA). The Revertaid first strand cDNA synthesis kit and SYBR green master mix were obtained from Thermo Scientific (Carlsbad, CA).

### Plant materials and preparation of M-BYF

*Epimedium brevicornu* (batch no. 180818), *Astragalus membranaceus* f. *pallidipurpureus* (batch no. 181225), *Rehmannia glutinosa* f. *lutea* (batch no. 180902), *Scutellaria baicalensis* f. *albiflora* (batch no. 181002) and *Paeonia lactiflora* var. *villosa* (batch no. 181102) were obtained from Shanghai Hongqiao Traditional Chinese Medicine Co., Ltd. (Shanghai, China). The voucher specimens were deposited in the Testing Centre of Shanghai Hongqiao Traditional Chinese Medicine Co., Ltd. (Shanghai, China). The herbs were botanically identified by researchers at Pharmacology Institute of Fudan University. The quality of these herbs was controlled by the Chinese Pharmacopoeia (The Stationery Office. 2015.). Briefly, the five herbal components of M-BYF were mixed in the ratio of 4:6:3:3:3 (dry weight) and soaked in distilled water (1:10 w/v) for 1 h. Then the mixture was boiled twice for 90 min at 100 °C. The decoctions were filtered, and the filtrate concentrated in a rotary evaporator at 65 °C. The percentage (w/w) yield of the water extract of M-BYF was approximately 50%. Before usage, M-BYF powder was dissolved in distilled water. According to the human and mice drug dose conversion formula, the doses of M-BYF used for mice intragastric administration per day were 7, 14 and 28 g/kg body weight.

### Chemical profiling of M-BYF using UPLC-Q-TOF-MS/MS

**Figure 1.**
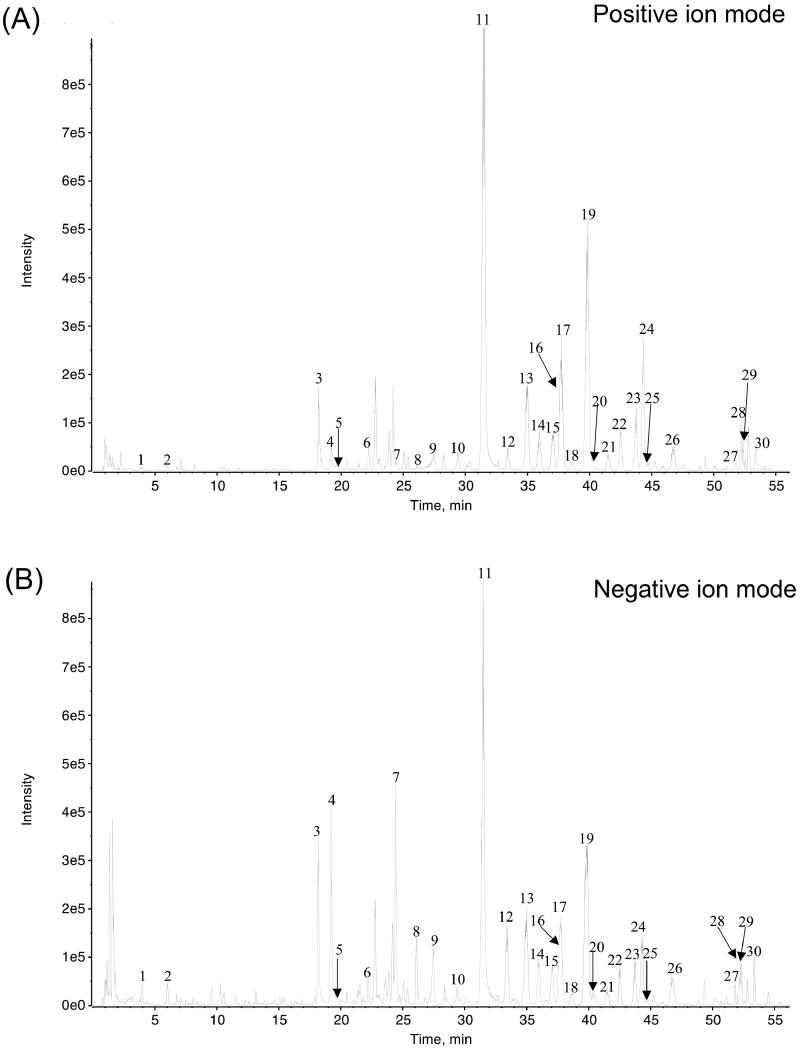
The UPLC-Q-TOF-MS/MS analysis carried out at both positive ion mode (A) and negative ion mode (B), and indicated 30 compounds in the M-BYF.

Ultra-performance liquid chromatography-quadrupole-time-of flight mass spectrometry (UPLC-Q-TOF-MS/MS) was utilized to qualitative analysis of major constituents of M-BYF. The analysis was performed with an Agilent 1290 LC system (Agilent, Palo Alto, CA) coupled to a SCIEX TripleTOF 4600^®^ quadrupole time-of-flight mass spectrometer (AB Sciex, Foster City, CA). Chromatographic separation was performed on an ACQUITY UPLC^®^ HSS T3 column (2.1 × 100 mm i.d., 1.8 μm; Waters, Milford, MA) at 25  C. The mobile phase was composed of 0.1% formic acid in both water (A) and acetonitrile (B). The gradient condition was used as follows: 0–5.0 min, 3% B; 5.0–10.0 min, 3–18% B; 10.0–20.0 min, 18–19.5% B; 20.0–37.0 min, 19.5–25% B; 37.0–47.0 min, 25–29% B; 47.0–58.0 min, 29–95% B; and 59 min, 95% B. The flow rate was 0.3 mL/min while the injection volume was 1 μL. The MS analysis was carried out in both positive and negative mode (Figure 1(A,B)). Total of 30 compounds were identified and showed in Table 1.

**Table 1.** Chemical profile of M-BYF.

No.	Retention time (min)	Adduct ion	*m/z* observed values	Compound name	Resource
1	3.97	[M + FA–H]^–^	421.1382	8-Debenzoylpaeoniflorin	*Paeonia lactiflora* var. *villosa*
2	6.02	[M + FA–H]^–^	407.1201	Catalpol	*Rehmannia glutinosa* f. *lutea*
3	18.19	[M + FA–H]^–^	525.1619	Albiflorin	*Paeonia lactiflora* var. *villosa*
4	19.22	[M + FA–H]^–^	525.1647	Paeoniflorin	*Paeonia lactiflora* var. *villosa*
5	19.73	[M–H]^–^	785.2523	Echinacoside	*Rehmannia glutinosa* f. *lutea*
6	22.24	[M + FA–H]^–^	491.1233	Calycosin-7-glucoside	*Astragalus membranaceus* f. *pallidipurpureus*
7	24.42	[M–H]^–^	623.2032	Acteoside	*Astragalus membranaceus* f. *pallidipurpureus*
8	26.12	[M–H]^–^	623.2032	Isoacteoside	*Astragalus membranaceus* f. *pallidipurpureus*
9	27.45	[M + H]^+^	525.1648	Isomer of paeoniflorin	*Paeonia lactiflora* var. *villosa*
10	29.4	[M–H]^–^	475.0917	Isomer of diosmetin-glucuronide	*Rehmannia glutinosa* f. *lutea*
11	31.49	[M–H]^–^	445.0794	Baicalin	*Scutellaria baicalensis* f. *albiflora*
12	33.41	[M–H]^–^	447.0973	Naringenin-7-*O*-glucuronide	*Astragalus membranaceus* f. *pallidipurpureus*
13	34.96	[M–H]^–^	445.0807	Norwogonin 7-*O*-β-d-glucuronide	*Scutellaria baicalensis* f. *albiflora*
14	35.97	[M–H]^–^	475.09	Isomer of diosmetin-glucuronide	*Rehmannia glutinosa* f. *lutea*
15	37.09	[M–H]^–^	445.0792	Norwogonin 8-*O*-β-d-glucuronide	*Scutellaria baicalensis* f. *albiflora*
16	37.63	[M–H]^–^	475.091	Diosmetin 7-*O*-β-d-glucuronide	*Rehmannia glutinosa* f. *lutea*
17	37.73	[M–H]^–^	459.0958	Oroxylin A 7-*O*-glucuronide	*Scutellaria baicalensis* f. *albiflora*
18	38.69	[M–H]^–^	445.0816	Baicalein 6-*O*-β-d-glucuronide	*Scutellaria baicalensis* f. *albiflora*
19	39.81	[M–H]^–^	459.0965	Wogonoside	*Scutellaria baicalensis* f. *albiflora*
20	40.26	[M + FA–H]^–^	883.2931	Hexandraside F	*Epimedium brevicornu* Maxim.
21	41.47	[M + FA–H]^–^	883.2911	Epimedin A	*Epimedium brevicornu* Maxim.
22	42.52	[M + FA–H]^–^	853.2866	Epimedin B	*Epimedium brevicornu* Maxim.
23	43.72	[M + FA–H]^–^	867.3011	Epimedin C	*Epimedium brevicornu* Maxim.
24	44.34	[M + FA–H]^–^	721.2374	Icariin	*Epimedium brevicornu* Maxim.
25	44.69	[M + FA–H]^–^	867.3007	Baohuoside VI	*Epimedium brevicornu* Maxim.
26	46.73	[M–H]^–^	269.048	Baicalein	*Scutellaria baicalensis* f. *albiflora*
27	51.8	[M–H]^–^	675.2349	Baohuoside VII	*Epimedium brevicornu* Maxim.
28	52.26	[M–H]^–^	645.2234	Baohuoside IV	*Epimedium brevicornu* Maxim.
29	52.36	[M–H]^–^	659.2415	2′-*O*-rhaMnosylicariside II	*Epimedium brevicornu* Maxim.
30	53.38	[M–H]^–^	513.1806	Baohuoside I	*Epimedium brevicornu* Maxim.

### Quantitation of main bioactive compounds from different batches of M-BYF using high-performance liquid chromatography (HPLC)

**Figure 2.**
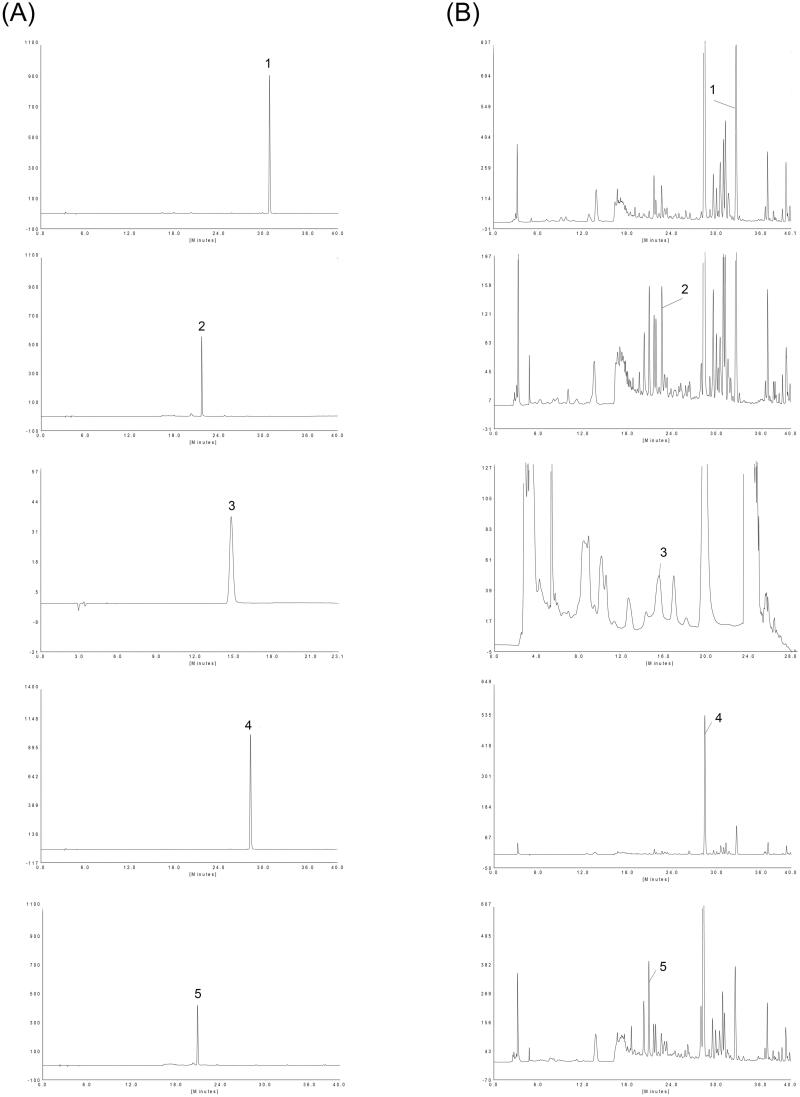
HPLC chromatograms of M-BYF at 270 nm. Representative chromatograms of standard reference compounds (A) and M-BYF (B) were shown respectively. (1) Icariin, (2) calycosin-7-glucoside, (3) catalpol, (4) baicalin and (5) paeoniflorin.

HPLC analysis was performed using an P230 series (Elite, Dalian, China). Chromatographic separation was performed on a Welch Ultimate XB-C18 column (4.6 × 250 mm i.d., 5 μm) maintained at 40 °C. A mobile phase consisting of water containing 0.3% phosphoric acid (A) and acetonitrile (B). A linear gradient elution was conducted as follows: 0–10 min, 2–4% B, 10–12 min, 4–20% B, 12–30 min, 20–40% B, 30–40 min, 40–95% B. The flow rate was 1 mL/min, and the total injection volume was 20 μL. Icariin, calycosin-7-glucoside, catalpol, baicalin and paeoniflorin were used as standard reference compounds. The typical chromatograms of the reference compounds and M-BYF are shown in Figure 2(A,B).

### Experimental animals and study design

Female BALB/c mice aged 5–6 weeks were purchased from Shanghai Jiesijie Laboratory Animal Co., Ltd. (Shanghai, China) and raised in the specific pathogen-free house of Fudan Medical Laboratory Animal Centre (Shanghai, China). The temperature of SPF house was 22 ± 2 °C and the relative humidity was 40–50%. Animals were given free access to sterile food and water and kept under a 12 h light/dark cycle. This study was conducted in accordance with the National Institutes of Health Guide for the Care and Use of Laboratory Animals. The protocol was approved by the Ethics Committee on Animal Experiments of School of Pharmacy, Fudan University (permission no. 2019-01-HSYY-DJC-01). Mice (*n* = 72) were randomly divided into the following six groups of 12 mice each: Control group, OVA-induced asthma model (Model) group, low-dose of M-BYF treated group (7 g/kg/d), middle-dose of M-BYF treated group (14 g/kg/d), high-dose of M-BYF treated group (28 g/kg/d) and dexamethasone treated group (1 mg/kg/d). The mouse asthma model was developed with the sensitization and challenge of OVA. In brief, on day 0 and 7, mice were sensitized with an intraperitoneal (i.p.) injection of 20 μg OVA (grade V) with 2 mg Alum in 0.2 mL phosphate-buffered saline (PBS). From day 14 to 16, mice were challenged with an aerosol of 3% OVA (grade II) solution for 30 min/day. Starting on day 21, OVA challenge was administered three times per week for four consecutive weeks. Mice in the Control group received the same volume of saline instead. From day 21 to 48, mice in the treated groups were orally administered with M-BYF or dexamethasone once a day, at doses of 7, 14 and 28 g/kg/d for M-BYF and 1 mg/kg/d for dexamethasone. Mice in the Control group and Model group received an equivalent volume of saline. Within 24 h of the last OVA challenge, mice were subjected to airway responsiveness evaluation.

### Acute oral toxicity test

Twenty-four BALB/c mice aged 5–6 weeks with weights of 18–23 g were randomly divided into four groups. Each group consisted of three mice of each sex. The test drug (M-BYF) was administered to mice intragastrically once a day or three times a day in the treated group at three dose levels of 10, 30 and 90 g/kg. Distilled water was administered to mice in the negative control group. The general symptoms, signs of toxicity and mortality were observed over 6 h after the initiation of administration, and once a day for seven days. The body weight of each mouse was measured before the treatment and seven days after treatment. The mortality was expressed as lethal dose (LD_50_), which was calculated by the method of Bliss (Bliss 1939).

### Measurement of airway responsiveness

Airway responsiveness including pulmonary resistance (*R*_L_) and dynamic pulmonary compliance (*C*_dyn_) in mice was detected by an invasive whole-body plethysmography (Buxco Electronics Inc., Troy, NY) as previously described (Wang, Wu et al. 2014). Briefly, within 24 h of the last OVA solution or saline challenge, mice were anaesthetized with pentobarbital sodium and underwent tracheal cannulation. Mice were then ventilated with a mechanical ventilator system and given aerosolized PBS and increasing doses of MCh (3.125, 6.25 and 12.5 mg/mL). The minimum values for *R*_L_ and *C*_dyn_ were calculated in response to increasing doses of MCh and expressed as a percentage change from the baseline value.

### Histopathological assessment

Lung tissues were fixed with 4% paraformaldehyde solution, and embedded in paraffin blocks. Thin sections (3–4 μm) were stained with haematoxylin and eosin (H&E), period acid-Schiff (PAS) and Masson’s trichrome, which were used to assess inflammatory cells infiltration, mucus secretion and collagen deposition, respectively. The severity of inflammatory cells infiltration in airway was estimated with a semi-quantitative scoring system: 0, no inflammatory cells infiltration; 1, a few inflammatory cells infiltration; 2, a ring of inflammatory cells infiltration, 1, cell layer deep; 3, a ring of inflammatory cells infiltration, 2–4 cells deep; 4, a ring of inflammatory cells infiltration, >4, cells deep (Tachdjian et al. 2010). The level of airway mucus secretion was represented as the ratio of the PAS-stained positive area (μm^2^) in the bronchus (*A*_PAS+_) to the perimeter (μm) of the basement membrane of bronchiole (*P*_bm_) (Tachdjian et al. 2010). The extent of collagen deposition was evaluated using a semi-quantitative scoring system: 0, no collagen in peribronchiolar space; 1, a thin layer of peribronchiolar collagen deposits; 2, a cluster of peribronchiolar collagen deposits; 3, a thick layer of peribronchiolar collagen deposits (Sulaiman et al. 2018). All slides were viewed in a light microscope (Nikon, Inc., Garden City, NY) and images were further quantified by Image-J software (NIH, Bethesda, MD).

### Flow cytometry

Lung tissues were digested with 200 U/mL DNase I and 1 mg/mL collagenase IV at 37 °C for 45 min. The suspension was filtered with a 40-μm cell strainer (BD Biosciences, San Diego, CA). After lysis of red blood cells with RBC lysis buffer (Thermo Fisher Scientific, Waltham, MA), cells were washed with PBS, pelleted by centrifugation and resuspended in PBS. For ILC2s staining, the single cell suspension was stained with the following fluorescein-conjugated monoclonal antibodies including APC-Cy7-conjugated Live/Dead, PE-Cy7-conjugated anti-CD3, anti-CD4, anti-CD19, anti-CD11b, eF506-conjugated anti-CD45, PE-conjugated anti-CD90.2, eF450-conjugated anti-CD127 and APC-conjugated anti-ST2. For Th9 cells staining, the single cell suspension was re-stimulated with leukocyte activation cocktail (BD Biosciences, San Diego, CA) and stained with APC-Cy7-conjugated Live/Dead, eF506-conjugated anti-CD45 and PE-Cy7-conjugated anti-CD4. After staining the surface markers, stimulated cells were fixed, permeabilized with a Foxp3 Perm/Fixation buffer and then stained with APC-conjugated anti-IL-9. Cell samples were detected on an Attune Nxt instrument (Thermo Fisher Scientific, Waltham, MA) and data were analysed with FlowJo software (version 10.4.1; Tree Star, Ashland, OR).

### Preparation and analysis of BALF

Bronchoalveolar lavage fluid (BALF) samples were collected from mice as previously described (Wang, Wu et al. 2014). The supernatants from BALF samples were collected by means of centrifugation at 1000×*g* for 15 min at 4 °C. The concentrations of IL-13, IL-5 and VIP in the supernatants were measured by ELISA according to the manufacturer’s instructions.

### RNA isolation and quantitative real-time PCR (qRT-PCR)

Total RNA was extracted from lung tissues of the mice using the RNAeasy kit (Beyotime, Shanghai, China) and then cDNA was generated using the Revertaid first strand cDNA synthesis kit (Thermo Fisher, Carlsbad, CA). qRT-PCR was performed on a StepOnePlus real-time PCR system (Applied Biosystems, Foster City, CA) with the following program settings: 95 °C for 5 min, 40 cycles at 95 °C for 10 s and 55 °C for 30 s. GAPDH was used as an internal control. The forward and reverse primer sequences are shown in Table 2. Data were obtained as cycle threshold (Ct) values and 2^−ΔΔCt^ was used in the analysis.

**Table 2.** qRT-PCR primer sequences.

Target gene	Primer sequence
GATA3-F	5′-CCAAGGCACGATCCAGCACAG-3′
GATA3-R	5′-TTATGGTAGAGTCCGCAGGCATTG-3′
IRF4-F	5′-AATGGTTGCCAGGTGACAGGAAC-3′
IRF4-R	5′-CGCCAAGGCTTCAGCAGACC-3′
PU.1-F	5′-TTCCAGTTCTCGTCCAAGCACAAG-3′
PU.1-R	5′-CTCGCCTGTCTTGCCGTAGTTG-3′
IL-9-F	5′-CAGCACCACATGGGGCATCAG-3′
IL-9-R	5′-TGGGACGGAGAGACACAAGCAG-3′
VIP-F	5′-GGAAGCCAGAAGCAAGCCTCAG-3′
VIP-R	5′-TCCTTCAAACGGCATCCTGTCATC-3′
GAPDH-F	5′-CAAGGCTGTGGGCAAGGTCATC-3′
GAPDH-R	5′-TCTCCAGGCGGCACGTCAG-3′

### Western blot analysis

Total proteins were extracted from lung tissues using RIPA buffer containing proteinase inhibitor. Protein concentrations were determined by the BCA protein assay method. The protein samples were separated on 10% sodium dodecyl sulphate-polyacrylamide gel electrophoresis and then transferred to the polyvinylidene difluoride membranes. The membranes were blocked with 5% non-fat milk in Tris-buffered saline and Tween 20 (TBS-T) for 1 h at room temperature. Then membranes were incubated overnight with the primary antibody at 4 °C. Antibodies against VPAC2 (1:1000; Affinity, Cincinnati, OH), GATA3, cAMP (1:1000, Proteintech, Wuhan, China), p-PKA substrate and β-actin (1:1000, Cell Signaling Technology, Danvers, MA) were used. The membranes were washed in TBS-T and incubated with horseradish peroxidase-conjugated secondary antibody for 1 h at room temperature. Immunoreactive bands were visualized by ECL detection (Millipore, Temecula, CA). Image J software (NIH, Bethesda, MD) was used to quantify the protein levels. β-Actin was used for standardization.

### Immunofluorescent assay

Lung sections were fixed with 4% paraformaldehyde at 4 °C and incorporated in 4% low-melting temperature agarose. Sections were then permeabilized with PBS containing 0.6% Triton X-100 and 2.5% bovine serum albumin for one day at room temperature. Staining procedure was performed by incubating sections with primary antibodies including anti-VIP, anti-CD90 and anti-VPAC2 antibodies. Samples were incubated for 1 h at room temperature with secondary antibodies and then counterstained for 10 min with 4′,6-diamidino-2-phenylindole (DAPI). A fluorescence microscope (NIKON ECLIPSE C1, Melville, NY) was used to assess positive staining and the percentages of VIP^+^ cells and VPAC2^+^CD90^+^ cells in lungs were quantified using the Image-J software (NIH, Bethesda, MD).

### Statistical analysis

Statistical analysis was performed using SPSS software (version 25, SPSS Inc., Chicago, IL). All of the values were represented as the mean ± standard error of mean (S.E.M.). One-way analysis of variance (ANOVA) followed by Bonferroni’s *post hoc* test or Dunnett’s test was used for statistical comparisons between groups. Differences were considered statistically significant when the *p* values <0.05 (two-tailed).

## Results

### UPLC-Q-TOF/MS analysis of bioactive compounds in M-BYF

Total ion chromatogram (TIC, positive mode and negative mode) and the identified compounds of M-BYF are shown in Figure 1(A,B) and Table 1. A total of 30 compounds were identified based on their adduct ions and characteristic MS/MS fragmental data, as well as previous literature data (Nurahmat et al. 2014; Tian et al. 2017; Shao et al. 2018).

### Quantification of five compounds in M-BYF

HPLC was applied to the quantitative analysis of three batches of M-BYF (Figure 2(A,B)). Contents of the five main bioactive compounds were determined in M-BYF and are summarized in Table 3. The content of baicalin was the highest, followed by paeoniflorin, icariin, catalpol and calycosin-7-glucoside. The relative standard deviation (RSD) values were all <10%. The content levels of the five constituents are stable in three sample batches of M-BYF.

**Table 3.** Contents of the five main compounds in three batches of M-BYF.

Compounds	Batch 1	Batch 2	Batch 3	Mean	RSD/%
Icariin	0.31%	0.35%	0.33%	0.33%	6.06
Calycosin-7-glucoside	0.043%	0.045%	0.051%	0.046%	8.99
Catalpol	0.178%	0.21%	0.179%	0.19%	9.63
Baicalin	3.92%	4.11%	3.77%	3.93%	4.33
Paeoniflorin	0.66%	0.74%	0.64%	0.68%	7.78

### Acute oral toxicity study of M-BYF in mice

**Figure 3.**
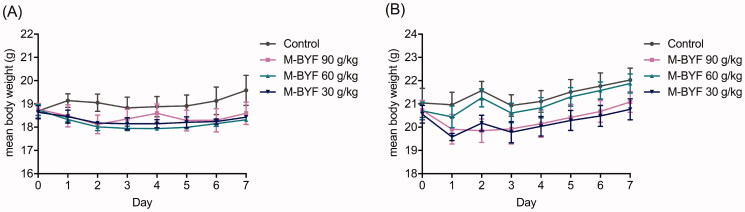
Changes in the body weight of mice treated with M-BYF for seven days. Time course of mean body weight after intragastric administration of M-BYF in female (A) and male (B) mice.

No mice died during the seven-day acute toxicity study. There was no significant difference in body weight of either female or male mice among the M-BYF treated group and the negative control group (Figure 3(A,B)). There were no abnormal signs regarding general appearance, behaviour, nervous system and clinical sings of adverse effects observed in mice treated with graded dose of M-BYF compared with mice treated with distilled water during the seven-day observation period. Based on these results, the medium lethal dose (LD_50_) value was estimated to be greater than 90 g/kg.

### M-BYF treatment reduced AHR in OVA-induced asthmatic mice

**Figure 4.**
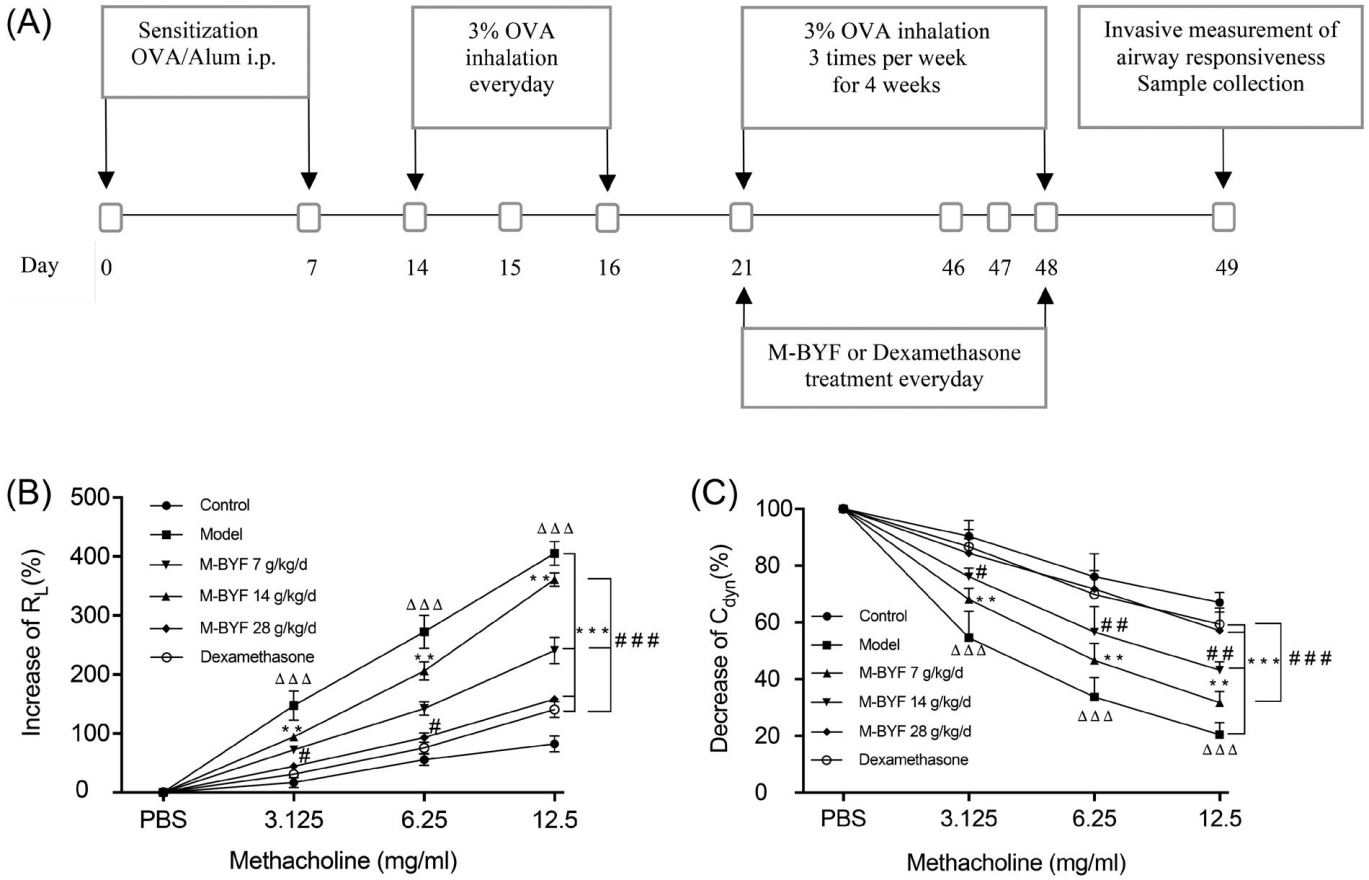
Experimental protocol of OVA sensitization and challenge in mice and effect of M-BYF on AHR. (A) Female BALB/c mice were sensitized and challenged with OVA in a 49-day asthma induction protocol. Starting on day 21, mice were treated with M-BYF or Dexamethasone by gavage every day during 28 days. Within 24 h of the last OVA challenge, anaesthetized mice were instrumented to measure AHR. (B) M-BYF markedly decreased *R*_L_ in asthmatic mice as compared with the Model group. (C) M-BYF obviously improved *C*_dyn_ in asthmatic mice as compared with the Model group. Dexamethasone was used as positive drug. Data are represented as mean ± S.E.M. *n* = 8 in each group. (^ΔΔΔ^*p* < 0.001 compared with the Control group; ***p < 0.001, **p < 0.01 compared with the Model group; ^###^*p* < 0.001, ^##^*p* < 0.01 and ^#^*p* < 0.05 compared with the dexamethasone treated group.)

On day 21 after the third OVA challenge, mice were treated with three different doses of M-BYF or dexamethasone for 28 days. Within 24 h of the last OVA challenge, mice were evaluated for airway responsiveness (Figure 4(A)). Mice in the Model group displayed significantly increased airway responsiveness to MCh, with a remarkable increase in *R*_L_ and a decrease in *C*_dyn_, as compared with the Control group (*p* < 0.001). Treatment with three different doses of M-BYF (7, 14 and 28 g/kg/d) and 1 mg/kg/d dose of dexamethasone significantly reduced OVA-induced AHR in asthmatic mice as compared with untreated asthmatic mice (*p* < 0.01 or 0.001). The inhibitory effect of the high-dose of M-BYF on *C*_dyn_ was similar to that of dexamethasone (*p* > 0.05) (Figure 4(B,C)). The result suggested that M-BYF played a significant role in the reduction in AHR.

#### M-BYF treatment alleviated airway inflammation, mucus hypersecretion and collagen deposition in OVA-induced asthmatic mice

**Figure 5.**
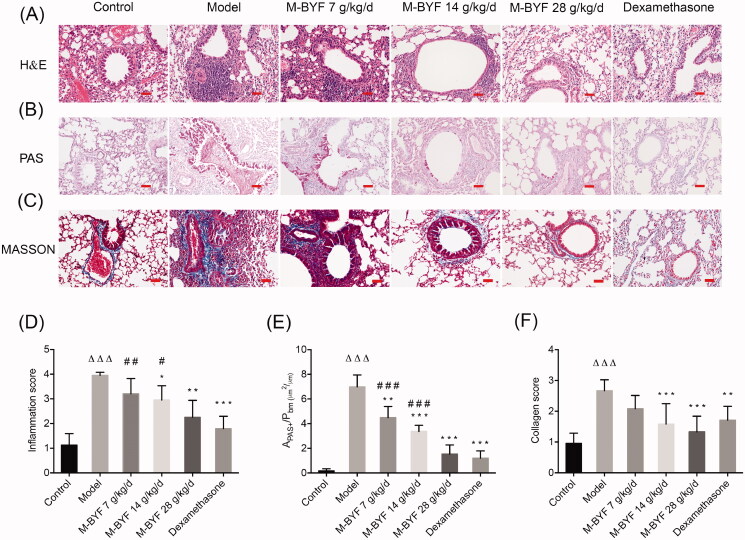
M-BYF alleviated airway inflammation, mucus hypersecretion and collagen deposition in OVA-induced asthmatic mice. (A) The infiltration of inflammatory cells into the lungs was measured by H&E staining. Scale bar: 50 µm. (D) The inflammatory changes were expressed as inflammation score. (B) Airway mucus secretion was detected by PAS staining. Scale bar: 50 µm. (E) Degree of mucus secretion was calculated by dividing PAS-stained positive area (μm^2^) in the bronchus (*A*_PAS+_) by the perimeter (μm) of the basement membrane (*P*_bm_). (C) Airway collagen deposition was evaluated by Masson’s trichrome staining. Scale bar: 50 µm. (F) The extent of collagen deposition was expressed as collagen score. Three non-consecutive sections from each animal were averaged and compared among experimental groups. *n* = 8 in each group. Data are represented as mean ± S.E.M. (^ΔΔΔ^*p* < 0.001 compared with the Control group; ***p < 0.001, **p < 0.01 and *p < 0.05 compared with the Model group; ^###^*p* < 0.001, ^##^*p* < 0.01 and ^#^*p* < 0.05 compared with the dexamethasone treated group.)

To further confirm the effects of M-BYF treatment on airway inflammation, mucus hypersecretion and collagen deposition, pathological examinations of lung sections were conducted using H&E, PAS and Masson’s trichrome staining methods. As compared with the Control group, a remarkable infiltration of inflammatory cells into the peribronchiolar and perivascular regions was observed in the Model group (*p* < 0.001). Treatment with the middle-dose and high-dose of M-BYF (14 and 28 g/kg/d) and 1 mg/kg/d dose of dexamethasone drastically decreased inflammatory cells infiltration in asthmatic mice compared to untreated asthmatic mice (*p* < 0.05, 0.01 or 0.001). The inhibitory effect of the high-dose of M-BYF on inflammatory cells infiltration was similar to that of dexamethasone (*p* > 0.05) (Figure 5(A,D)). Furthermore, mice in the Model group exhibited a significant mucus hypersecretion in bronchial mucosa and airway lumen compared to the Control group (*p* < 0.001). In contrast, mice treated with three different doses of M-BYF (7, 14 and 28 g/kg/d) and 1 mg/kg/d dose of dexamethasone displayed a remarkable decrease of mucus secretion (*p* < 0.01 or 0.001). The inhibitory effect of the high-dose of M-BYF on mucus hypersecretion was similar to that of dexamethasone (*p* > 0.05) (Figure 5(B,E)). Airway collagen deposition was significantly increased in the Model group, as compared to the Control group (*p* < 0.001). The middle-dose and high-dose of M-BYF (14, 28 g/kg/d) treatment remarkably decreased collagen deposition in asthmatic mice, as compared with untreated asthmatic mice (*p* < 0.001). The effects of three different doses of M-BYF on decrease of collagen deposition were similar to that of dexamethasone (*p* > 0.05) (Figure 5(C,F)). These findings demonstrated that pathophysiological abnormalities in the mouse asthma model were significantly alleviated by M-BYF treatment. M-BYF treatment reduced percentages of ILC2s and Th9 cells in lungs of OVA-induced asthmatic mice.

#### M-BYF treatment reduced percentages of ILC2s and Th9 cells in lungs of OVA-induced asthmatic mice

**Figure 6.**
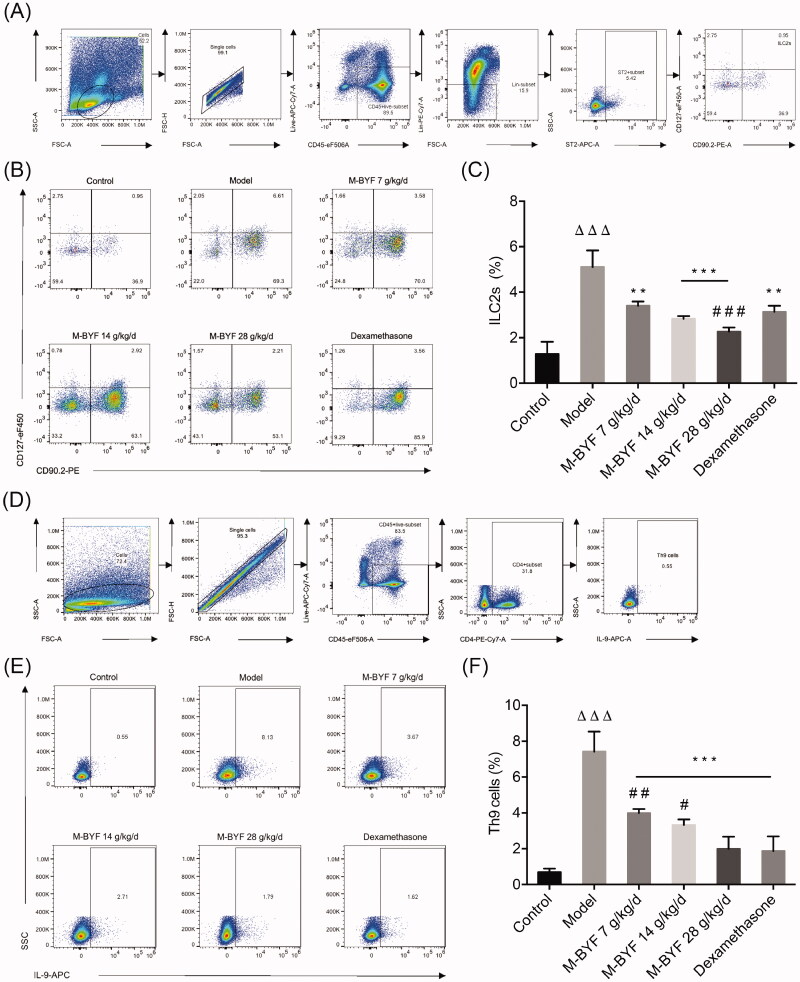
Effects of M-BYF on percentages of ILC2s and Th9 cells in lungs of OVA-induced asthmatic mice. (A) Gating strategy of ILC2s in the lungs of mice. Lineage (Lin) markers included CD3, CD19, CD4 and CD11b. The number indicates cell events. (B) Detection of ILC2s (CD45^+^Lin^–^ST2^+^CD90.2^+^CD127^+^) by flow cytometry. (C) M-BYF reduced percentage of ILC2s in lungs of asthmatic mice as compared with the Model group. (D) Gating strategy of Th9 cells in the lungs of mice. The number indicates cell events. (E) Detection of Th9 cells (CD45^+^CD4^+^IL9^+^) by flow cytometry. (F) M-BYF reduced percentage of Th9 cells in lungs of asthmatic mice as compared with the Model group; *n* = 8 in each group. Data are represented as mean ± S.E.M. (^ΔΔΔ^*p* < 0.001 compared with the Control group; ***p < 0.001, **p < 0.01 compared with the Model group; ^###^*p* < 0.001, ^##^*p* < 0.01 and ^#^*p* < 0.05 compared with the dexamethasone treated group.)

ILC2s and Th9 cells act as central initiators and amplifiers of type 2 immune responses in asthma (von Moltke and Pepper 2018). They play cooperative roles in many of the pathophysiological abnormalities characterizing asthma (Ying et al. 2016; Koch et al. 2017; Moretti et al. 2017). To further evaluate whether M-BYF has regulatory effects on ILC2s and Th9 cells, the percentages of these cells in the lungs were determined by flow cytometric analysis. ILC2s were defined as CD45^+^Lineage (Lin)^–^ST2^+^CD90.2^+^CD127^+^ cells. Th9 cells were defined as CD45^+^CD4^+^IL-9^+^ cells. Gating strategies of lung ILC2s and Th9 cells are shown in Figure 6(A,D). The percentages of lung ILC2s and Th9 cells were significantly increased in the Model group when compared to the Control group (*p* < 0.001) (Figure 6(B,C,E,F)). Treatment with three different doses of M-BYF (7, 14 and 28 g/kg/d) and 1 mg/kg/d dose of dexamethasone significantly reduced the percentages of ILC2s and Th9 cells in the lungs of asthmatic mice, as compared with untreated asthmatic mice (*p* < 0.01 or 0.001). There was no significant difference in the percentage of Th9 cells between the high-dose of M-BYF (28 g/kg/d) treated group and dexamethasone treated group (*p* > 0.05). Moreover, the effect of high-dose of M-BYF on decrease of ILC2s appeared to be stronger than that of dexamethasone (*p* < 0.001) (Figure 6(B,C,E,F)).

#### M-BYF treatment decreased expression of type 2 cytokines and GATA3, PU.1, IRF4 in OVA-induced asthmatic mice

**Figure 7.**
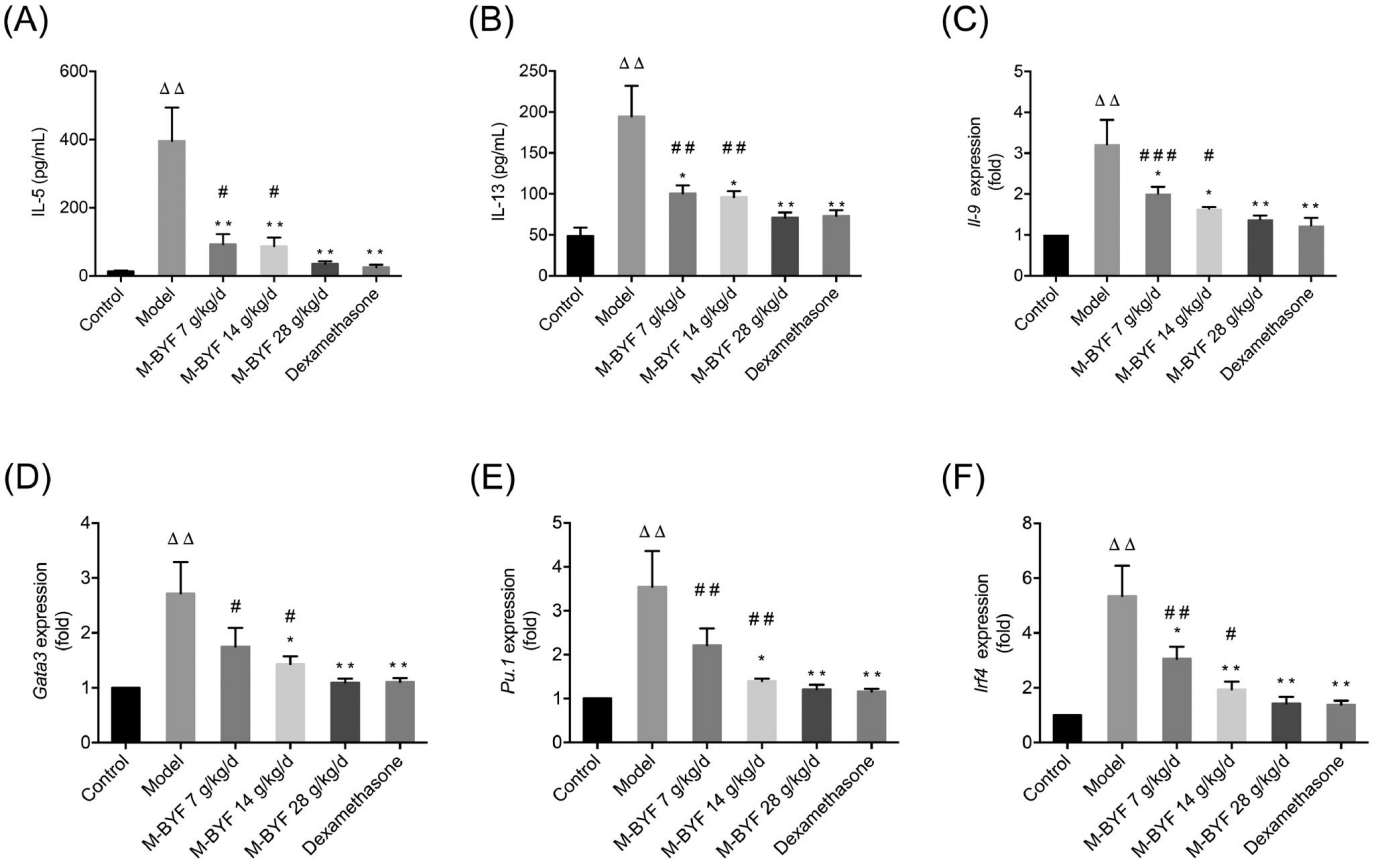
Effects of M-BYF on expression of IL-5, IL-13, IL-9, GATA3, PU.1 and IRF4 in OVA-induced asthmatic mice. M-BYF decreased levels of IL-5 (A), IL-13 (B) in BALF of asthmatic mice as compared with the Model group. M-BYF decreased gene expression of IL-9 (C), GATA3 (D), PU.1 (E) and IRF4 (F) in lungs of asthmatic mice as compared with the Model group; *n* = 6 in each group. Data are represented as mean ± S.E.M. (^ΔΔ^*p* < 0.01 compared with the Control group; ***p < 0.001, **p < 0.01 and *p < 0.05 compared with the Model group; ^###^*p* < 0.001, ^##^*p* < 0.01 and ^#^*p* < 0.05 compared with the dexamethasone treated group.)

Tissue resident ILC2s are the dominant early source of the canonical type 2 cytokines including IL-5, IL-13 and IL-9 in the lung (Halim 2016). Th9 cells are also the main source of IL-9 *in vivo* (Koch et al. 2017). IL-5, IL-13 and IL-9 production by activated ILC2 cells and Th9 cells in response to allergens induce type 2 inflammation and promote cardinal pathophysiological abnormalities in asthma (von Moltke and Pepper 2018). As shown in Figure 7(A–C), after OVA challenge, the levels of IL-5, IL-13 and IL-9 were significantly increased in BALF or lungs of mice (*p* < 0.01). Lower IL-5, IL-13 levels in the BALF and lower IL-9 gene expression in the lungs were observed in the M-BYF treated groups (7, 14 and 28 g/kg/d) and dexamethasone treated group compared to the Model group (*p* < 0.05 or 0.01). This result confirmed the attenuation of type 2 inflammation in asthmatic mice treated with M-BYF and dexamethasone. The suppressive effects of the high-dose of M-BYF (28 g/kg/d) on IL-5, IL-13 and IL-9 levels were similar to that of dexamethasone (*p* > 0.05). Moreover, we assessed the role of M-BYF on mRNA expression of transcription factors (TFs) including GATA3, PU.1 and interferon regulatory factor 4 (IRF4), which are required for the differentiation and function of ILC2s and Th9 cells. The mRNA expression of GATA3, PU.1 and IRF4 was significantly increased in the lungs of asthmatic mice compared with the normal mice (*p* < 0.01) (Figure 7(D–F)). Treatment with the middle-dose and high-dose of M-BYF (14 and 28 g/kg/d) and dexamethasone decreased GATA3, PU.1 and IRF4 mRNA expression in the lungs of asthmatic mice compared to untreated asthmatic mice (*p* < 0.05 or 0.01). The low-dose of M-BYF (7 g/kg/d) treatment also induced a decrease of IRF4 mRNA expression in the lungs of asthmatic mice compared to untreated asthmatic mice (*p* < 0.05). The inhibitory effects of the high-dose of M-BYF on GATA3, PU.1 and IRF4 mRNA levels were similar to that of dexamethasone (*p* > 0.05). Collectively, these results indicated that M-BYF treatment could alleviate type 2 airway inflammation and pathophysiological abnormalities in the mouse asthma model through the negative regulation of ILC2s and Th9 cells expansion/differentiation and function.

### M-BYF treatment decreased VIP expression in OVA-induced asthmatic mice

**Figure 8.**
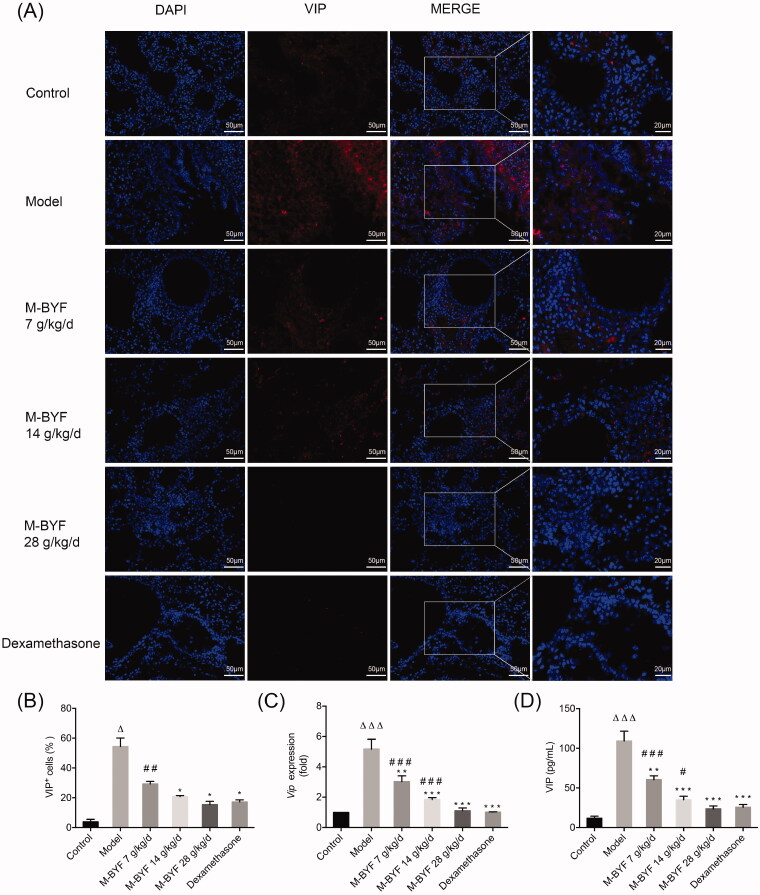
Effects of M-BYF on VIP expression and percentage of VIP^+^ cells in OVA-induced asthmatic mice. (A) VIP was down-regulated in the lungs of asthmatic mice with treatment of M-BYF by immunofluorescent staining. VIP staining as shown in red. Scale bar: 50 µm and 20 µm. (B) M-BYF decreased percentage of VIP^+^ cells in lungs of asthmatic mice as compared with the Model group. Four non-consecutive sections from each animal were averaged and compared among experimental groups; *n* = 3 in each group. M-BYF decreased gene expression of VIP (C) in lungs and level of VIP (D) in BALF of asthmatic mice as compared with the Model group; *n* = 6 in each group. Data are represented as mean ± S.E.M. (^ΔΔΔ^*p* < 0.001, ^Δ^*p* < 0.05 compared with the Control group; ***p < 0.001, **p < 0.01 and *p < 0.05 compared with the Model group; ^###^*p* < 0.001, ^##^*p* < 0.01 and ^#^*p* < 0.05 compared with the dexamethasone treated group.)

It has been reported that neuropeptide VIP induces and promotes type 2 immune responses by activating ILC2s and T cells as well as by increasing type 2 cytokines and TF GATA3 expression (Jimeno et al. 2012; Talbot et al. 2015). To explore the effect of M-BYF on VIP expression, we performed ELISA, qRT-PCR and immunofluorescent assay to measure the VIP expression levels and percentage of VIP^+^ cells in lungs or BALF of mice. As shown in Figure 8(A–D), VIP expression and percentage of VIP^+^ cells were increased after OVA-challenge (*p* < 0.05 or 0.001), but lower expression levels were observed in the middle-dose and high-dose of M-BYF (14 and 28 g/kg/d) and dexamethasone treated groups compared to the Model group (*p* < 0.05 or 0.001). The low-dose of M-BYF (7 g/kg/d) also significantly decreased VIP level in BALF and VIP mRNA expression in lungs of asthmatic mice compared to untreated asthmatic mice (*p* < 0.01). The inhibitory effect of the high-dose of M-BYF on VIP expression was similar to that of dexamethasone (*p* > 0.05). Meanwhile, there was no significant difference in percentage of VIP^+^ cells among middle-dose and high-dose of M-BYF treated groups and dexamethasone treated group (*p* > 0.05).

### M-BYF treatment decreased VPAC2 expression in OVA-induced asthmatic mice

**Figure 9.**
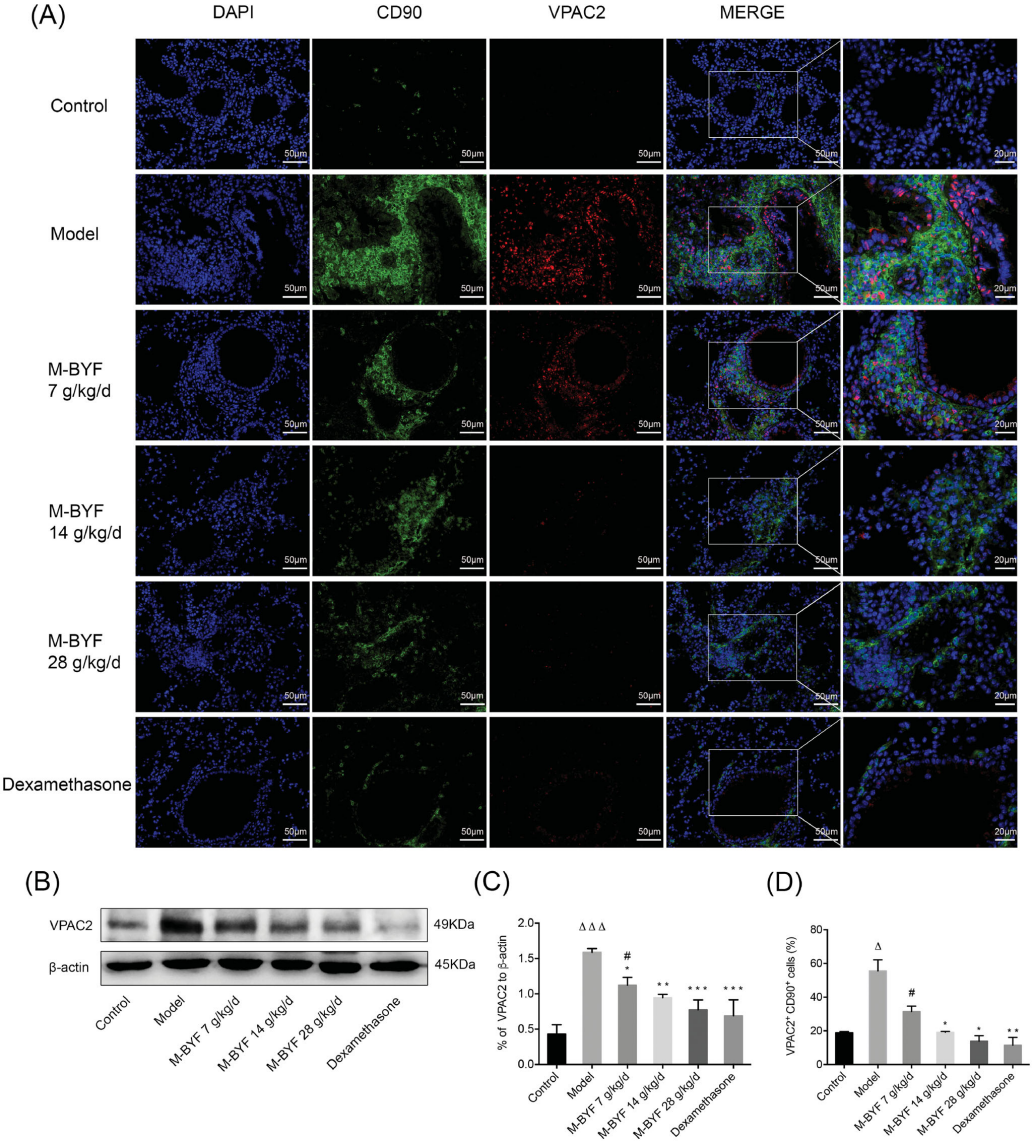
Effects of M-BYF on VPAC2 expression and VPAC2^+^CD90^+^ cells in OVA-induced asthmatic mice. (A) Representative immunofluorescent staining images of VPAC2 and VPAC2^+^CD90^+^ cells. VPAC2 staining as shown in red. CD90 staining as shown in green. Scale bar: 50 µm and 20 µm. (B, C) The protein expression of VPAC2 in lungs was detected by western blot and densitometric analysis was performed. M-BYF reduced the expression of VPAC2 protein in lungs of asthmatic mice as compared with the Model group (D) Quantification of VPAC2^+^CD90^+^ cells showed that M-BYF reduced percentage of VPAC2^+^CD90^+^ cells in lungs of asthmatic mice as compared with the Model group. Four non-consecutive sections from each animal were averaged and compared among experimental groups; *n* = 3 in each group. Data are represented as mean ± S.E.M. (^ΔΔΔ^*p* < 0.001, ^Δ^*p* < 0.05 compared with the Control group; ***p < 0.001, **p < 0.01 and *p < 0.05 compared with the Model group; ^#^*p* < 0.05 compared with the dexamethasone treated group.)

VIP promotes type 2 immune response when signalling through its G protein-coupled receptor, VPAC2, which is the dominant transducer of effects of VIP on activated Th cells and ILC2s (Voice et al. 2003; Delgado and Ganea 2013; Nussbaum et al. 2013; Talbot et al. 2015). To test whether M-BYF affects VPAC2 expression in mice, western blot analyses and immunofluorescence assay were performed to detect VPAC2 expression in the lungs. As shown in Figure 9(A–C), VPAC2 expression was increased in the Model group, as compared with the Control group (*p* < 0.001). M-BYF treatment and dexamethasone treatment significantly decreased VPAC2 expression in asthmatic mice compared to untreated asthmatic mice (*p* < 0.05, 0.01 or 0.001). The inhibitory effects of the middle-dose and high-dose of M-BYF (14 and 28 g/kg/d) on VPAC2 expression were similar to that of dexamethasone (*p* > 0.05). These results indicated that M-BYF inhibited VIP–VPAC2 signalling.

### M-BYF treatment decreased percentage of VPAC2^+^CD90^+^ cells in OVA-induced asthmatic mice

It has been demonstrated that VPAC2 receptor is expressed mainly in activated immune cells including T cells, macrophages and ILC2s (Delgado and Ganea 2013). VPAC2 expression in T cells was induced following stimulation of the antigen, T cell receptor, type 2 cytokines and VIP (Delgado et al. 1999; Dorsam et al. 2000; Goetzl et al. 2001; Nussbaum et al. 2013). CD90 is particularly abundant on the surface of mouse thymocytes and peripheral T cells (Haeryfar and Hoskin 2004). Lung resident ILC2s also expressed CD90 (Monticelli et al. 2011). To explore whether the expansion/differentiation of ILC2s and T cells in the lungs of asthmatic mice might be associated with increased expression of VPAC2, we stained lung sections with anti-VPAC2 and anti-CD90 antibodies. The percentage of VPAC2^+^CD90^+^ cells in lungs was significantly increased in the Model group, as compared with the Control group (*p* < 0.05). The middle-dose and high-dose of M-BYF (14 and 28 g/kg/d) treatment and dexamethasone treatment observably decreased the percentage of VPAC2^+^CD90^+^ cells in lungs of asthmatic mice (*p* < 0.05 or 0.01) (Figure 9(A,D)). The data indicated that VIP–VPAC2 signalling might be involved in M-BYF-mediated repression of ILC2s and T cells expansion/differentiation.

### M-BYF treatment inhibited VPAC2-cAMP-PKA-GATA3 signalling pathway in OVA-induced asthmatic mice

**Figure 10.**
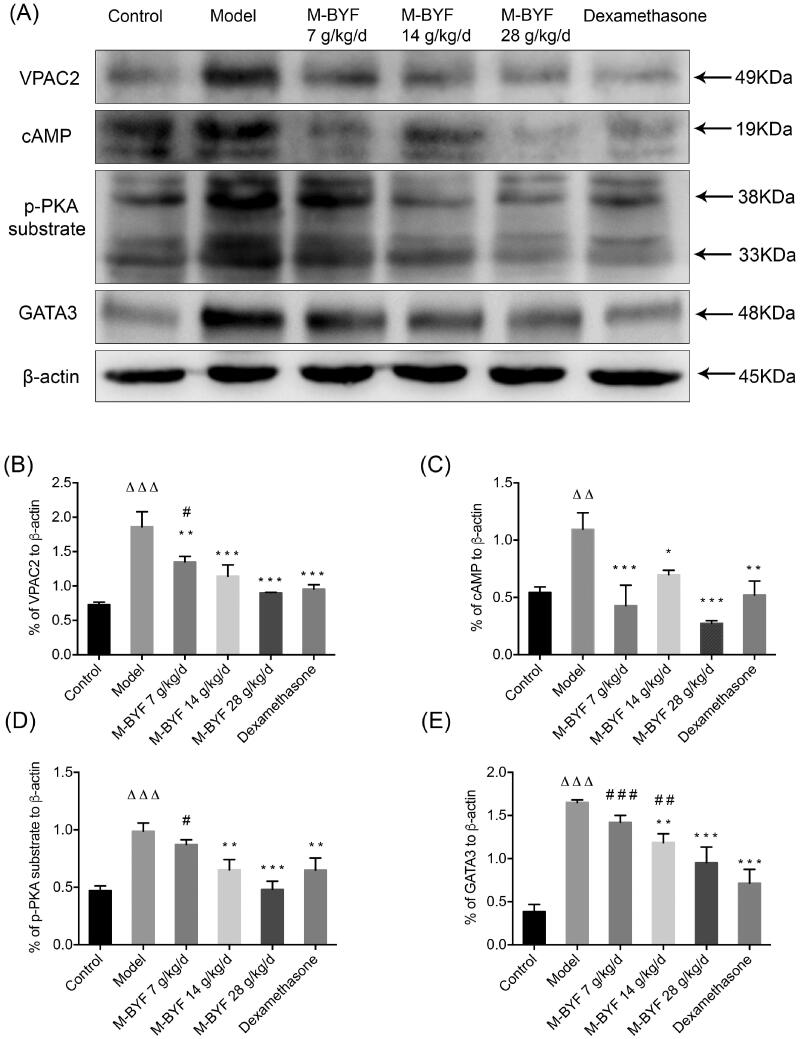
Effects of M-BYF on protein expression of VPAC2, cAMP, p-PKA substrate and GATA3 in lungs of OVA-induced asthmatic mice. (A) Western blot analysis of the protein expression of VPAC2, cAMP, p-PKA substrate and GATA3. (B–E) Quantification of relative levels of VPAC2, cAMP, p-PKA substrate and GATA3 showed that M-BYF reduced the expression of proteins associated with the VIP–VPAC2 signalling pathway in lungs of asthmatic mice as compared with the Model group; *n* = 3 in each group. Data are represented as mean ± S.E.M. (^ΔΔΔ^*p* < 0.001, ^ΔΔ^*p* < 0.01 compared with the Control group; ***p < 0.001, **p < 0.01 and *p < 0.05 compared with the Model group; ^###^*p* < 0.001, ^##^*p* < 0.01 and ^#^*p* < 0.05 compared with the dexamethasone treated group.)

We next investigated the signalling cues provided by activated VPAC2 in lung tissues. As shown in Figure 10(A–E)), expression of VPAC2, cAMP, p-PKA substrate and GATA3 proteins was significantly increased in lungs of mice after OVA challenge (*p* < 0.01 or 0.001). The middle-dose and high-dose of M-BYF (14 and 28 g/kg/d) treatment and dexamethasone treatment led to impaired VPAC2, cAMP, p-PKA substrate and GATA3 expression in asthmatic mice compared to untreated asthmatic mice (*p* < 0.05, 0.01 or 0.001). The low-dose of M-BYF (7 g/kg/d) also significantly decreased expression of VPAC2 and cAMP (*p* < 0.01 or 0.001) (Figure 10(A–E)). These results indicated that inhibition of VPAC2-cAMP-PKA-GATA3 signalling pathway might be involved in the regulatory effects of M-BYF on differentiation and activation of ILC2s and T cells.

## Discussion

The mouse model of OVA-induced asthma is one of the most extensively used models characterized by aberrant type 2 inflammation and eosinophilia (Gold et al. 2015). In the present study, this model was established to evaluate the protective effects of M-BYF against allergic asthma. The results demonstrated that M-BYF alleviated allergic airway inflammation, AHR, mucus hypersecretion and collagen deposition in the mouse asthma model. These results are consistent with the previous observation that BSYQF treatment could attenuate pathological changes in OVA-induced asthmatic mice (Wang, Wu et al. 2014). Moreover, we showed for the first time that M-BYF treatment decreased percentages of ILC2s and Th9 cells in the lungs of asthmatic mice, which are associated with the inhibition of allergic airway inflammation. Meanwhile, M-BYF treatment decreased expression of TFs including GATA3, IRF4 and PU.1 in lungs of asthmatic mice. IRF4, PU.1 and GATA3 are crucial for Th9 cells differentiation and IL-9 production (Kaplan et al. 2015). GATA3 is also essential for development and function of ILC2s, and has been shown to contribute to type 2 inflammation through directly transactivating the type 2 cytokine genes and inducing type 2 cytokines production in activated ILC2s, Th9 cells and Th2 cells (Klein Wolterink et al. 2013; Tindemans et al. 2014). Consistent with our observation in ILC2s, Th9 cells and their master TFs, the expression of type 2 cytokines including IL-5, IL-13 and IL-9 in asthmatic mice was decreased after treatment of M-BYF. These results suggested that M-BYF treatment alleviated type 2 airway inflammation and pathophysiological abnormalities, probably by negative regulation of lung ILC2s and Th9 cells differentiation and function. While ILC2s and some subsets of T cells have been demonstrated to induce and promote type 2 immune responses in allergic asthma, the regulatory mechanisms contribute to activation and differentiation of them in type 2 airway inflammation are multifactorial and complex. To further explore the mechanism of M-BYF-mediated suppression of type 2 inflammation, we investigated the role of VIP, which has potent effects on the proliferation, differentiation and function of T cells and ILC2s (Voice et al. 2001; Jimeno et al. 2012). VIP is a member of neuroendocrine and immune peptide mediators that supplied to peripheral organs like the lung by an abundant supply of synapsing nerves. Additionally, upon antigenic stimulation, immune cells such as T cells, ILC2s and macrophages contribute to the local production of VIP in the lungs and respond to VIP in an autocrine/paracrine manner (Samarasinghe et al. 2011; Nussbaum et al. 2013). The role that VIP plays in asthma is highly dependent on resident immune cell populations, surrounding microenvironment of the organ and the presence of specific VIP associated receptor (Verma et al. 2017). VIP promotes type 2 immune responses by signalling through VPAC2, which is highly expressed on activated T cells, and ILC2s (Goetzl et al. 2001; Nussbaum et al. 2013). It has been demonstrated that the transgenic mice with high constitutive VPAC2 expression in T cells showed immediate-type reactions to allergy-producing antigens while VPAC2 receptor genetic deletion or knockout mouse has depressed allergic reactions (Goetzl et al. 2001; Voice et al. 2001). Recently, it was reported that VIP expression was increased in BALF and lung sensory neurons of OVA-exposed mice compared to naïve mice. VIP–VPAC2 signalling activated ILC2s and CD4^+^ T cells to produce type 2 cytokines, resulting in amplification of type 2 inflammation in allergic asthma (Talbot et al. 2015). Consistent with previous studies, our results showed that VIP and VPAC2 expression was higher in OVA-induced asthmatic mice compared with normal mice. Treatment with M-BYF decreased the VIP and VPAC2 expression in asthmatic mice. Meanwhile, we found that M-BYF treatment decreased percentage of VPAC2^+^CD90^+^ cells in the lungs of asthmatic mice. Previous research has reported that in human Jurkat T cell line and CD4^+^T cells, activation of VPAC2 leads to increase of cAMP and activation of cAMP-dependent PKA (Delgado et al. 2000; Grinninger et al. 2004). In CD4^+^T cells and Th9 cells, activation of cAMP-PKA pathway induces type 2 cytokines such as IL-5 gene expression, IL-9 production in a GATA3-dependent manner (Klein-Hessling et al. 2003; Mikami et al. 2013). Importantly, GATA3 acts both as a TF continuously expressed by ILC2s and as being essential for differentiation and maintenance of ILC2s and Th9 cells (Goswami et al. 2012; Hoyler et al. 2012). In our previous research, we found that BuShenYiQi formula could decrease expression level of GATA3 in CD4^+^T cells which were isolated from OVA-sensitized mice (Wang, Wu et al. 2014). Therefore, we analysed the VPAC2-cAMP-PKA-GATA3 pathway in OVA-induced asthmatic mice after treatment with M-BYF by western blotting. We demonstrated that expression of VPAC2, cAMP, p-PKA substrate and GATA3 was significantly increased in the lungs of mice after OVA challenge. M-BYF treatment decreased pulmonary expression of VPAC2, cAMP, p-PKA substrate and GATA3 in asthmatic mice. These data further demonstrated that inhibition of VIP–VPAC2–cAMP–PKA–GATA3 signalling pathway might be involved in M-BYF-mediated regulation of differentiation and function of ILC2s and Th9 cells.

Based on these results, we deduced that M-BYF inhibition of type 2 inflammation in OVA-induced asthmatic mice partly depended on negative regulation of VIP–VPAC2 signalling pathway and differentiation/expansion and function of ILC2s and T cells. However, this is should be further confirmed by blocking VPAC2 receptor or adopting VPAC2 mutant mice in the further study. In this study, we also determined the chemical compositions of M-BYF. The UPLC-Q-TOF-MS/MS analysis showed that there are abundant glycosides and flavonoids and some iridoid glycosides in the M-BYF, such as baicalin, icariin, paeoniflorin, albiflorin and catalpol. Baicalin is a well-studied natural monomer in *Scutellaria baicalensis* f. *albiflora* with promising anti-asthmatic and anti-remodelling effects in OVA-induced mouse model of asthma (Sun et al. 2013; Ma et al. 2014). Baicalin could inhibit type 2 airway inflammation, and downregulate the enhancement of type 2 cytokines including IL-5, IL-13 in mast cell (Yoshida et al. 2021). Moreover, baicalin could profoundly decrease neuropeptide calcitonin gene-related peptide (CGRP) level to alleviate pain responses of migraine rats. Baicalin has also been established to have neuroprotective properties (Jin et al. 2019). Icariin is a major bioactive monomer in *Epimedium brevicornu* and has been applied in treating autoimmune diseases such as asthma due to its anti-inflammatory activity. Icariin has also been demonstrated to restore neuronal endoplasmic reticulum stress in asthmatic mice (Liu et al. 2019).

Previous research reported the paeoniflorin and albiflorin which were derived from *Paeonia lactiflora* var. *villosa*, possessed good anti-inflammatory and neuroprotective activities (Wang, Gao et al. 2014). Catalpol, derived from *Rehmannia glutinosa* f. *lutea*, has been shown to have neuroprotective effects, antioxidant and anti-inflammatory potential for intervening various diseases (Huang et al. 2013). Catalpol could inhibit the expression of IL-13, IL-5 and reduce eosinophils infiltration in the lung to inhibit OVA-induced allergic airway inflammation (Chen et al. 2017; Zhu and Wang 2019). In addition, catalpol could also improve the endocrine function of the hypothalamic-pituitary-adrenocortical-axis (HPA) and inhibit neuropeptide endothelin-1 (ET-1) to exert neuroprotective effects (Liu et al. 2014; Wang, Wu et al. 2014; Wang, Gao et al. 2014; Wang JH et al. 2014). Meanwhile, quantitative analysis of M-BYF showed that baicalin exhibited the highest concentrations, followed by paeoniflorin, icariin, catalpol and calycosin-7-glucoside.

Taken together, we deduced that the pharmacological actions and effectiveness of M-BYF in inhibiting type 2 airway inflammation and regulating neuropeptide signalling might be mainly attributed to anti-inflammatory and neuroprotective properties of flavonoids baicalin. Importantly, many chemical compositions of M-BYF such as icariin, epimedin C and catalpol have similar effects but act through different mechanisms to alleviate type 2 airway inflammation and pathophysiological abnormalities in allergic asthma. These bioactive compounds may produce additive or synergistic effects against asthma. It might be worth to explore the mechanisms of different chemical compositions of M-BYF for the treatment of allergic asthma and to identify key bioactivity-related chemical components within M-BYF in the future study.

## Conclusions

M-BYF treatment significantly alleviated type 2 airway inflammation, AHR, mucus hypersecretion and collagen deposition in experimental allergic asthma, which was related to the decrease of expansion/differentiation and activities of ILC2s and Th9 cells, as well as to the inhibition of VIP–VPAC2 signalling.
